# Lumican delays melanoma growth in mice and drives tumor molecular assembly as well as response to matrix-targeted TAX2 therapeutic peptide

**DOI:** 10.1038/s41598-017-07043-9

**Published:** 2017-08-09

**Authors:** Albin Jeanne, Valérie Untereiner, Corinne Perreau, Isabelle Proult, Cyril Gobinet, Camille Boulagnon-Rombi, Christine Terryn, Laurent Martiny, Stéphane Brézillon, Stéphane Dedieu

**Affiliations:** 1 Université de Reims Champagne-Ardenne, UFR Sciences Exactes et Naturelles, Campus Moulin de la Housse, 51100 Reims, France; 2 CNRS UMR 7369, Matrice Extracellulaire et Dynamique Cellulaire, MEDyC, 51 rue Cognacq Jay, 51100 Reims, France; 3 SATT Nord, 25 avenue Charles Saint-Venant, 59800 Lille, France; 4 Plateforme d’Imagerie Cellulaire et Tissulaire, Université de Reims Champagne-Ardenne, 51 rue Cognacq Jay, 51100 Reims, France; 5 Université de Reims Champagne-Ardenne, UFR Médecine, 51 rue Cognacq Jay, 51100 Reims, France; 6 Université de Reims Champagne-Ardenne, UFR Pharmacie, 51 rue Cognacq Jay, 51100 Reims, France; 7 CHU de Reims, Laboratoire Central d’Anatomie et de Cytologie Pathologique, 51100 Reims, France

## Abstract

Lumican is a small leucine-rich proteoglycan (SLRP) being known as a key regulator of collagen fibrillogenesis. However, little attention has been given so far in studying its influence on tumor-associated matrix architecture. Here, we investigate the role of host lumican on tumor matrix organization as well as on disease progression considering an immunocompetent model of melanoma implanted in *Lum* ^−/−^ *vs*. wild type syngeneic mice. Conjointly, lumican impact on tumor response to matrix-targeted therapy was evaluated considering a previously validated peptide, namely TAX2, that targets matricellular thrombospondin-1. Analysis of available genomics and proteomics databases for melanoma first established a correlation between lumican expression and patient outcome. In the B16 melanoma allograft model, endogenous lumican inhibits tumor growth and modulates response to TAX2 peptide. Indeed, IHC analyses revealed that lumican deficiency impacts intratumoral distribution of matricellular proteins, growth factor and stromal cells. Besides, innovative imaging approaches helped demonstrating that lumican host expression drives biochemical heterogeneity of s.c. tumors, while modulating intratumoral collagen deposition as well as organization. Altogether, the results obtained present lumican as a strong endogenous inhibitor of tumor growth, while identifying for the first time this proteoglycan as a major driver of tumor matrix coherent assembly.

## Introduction

During cancer progression, intricate cross-talk is established between malignant cells and a tumor-specific microenvironment consisting of both stromal cells and extracellular matrix (ECM). While tumor ECM is continuously submitted to intense remodeling, dysregulation of its integrity crucially contributes to the severity of disease progression^1^. Therefore, numerous promising anticancer strategies that focus on ECM macromolecules have been developed over the last years^2, 3^. This may include either interfering with matrix biosynthesis as well as self-assembly^4^ or targeting matricellular proteins, i.e. non-structural elements that regulate cell-ECM communication and cancer cell behavior^5^.

Among the molecules that control stromal collagen matrix assembly, lumican is a small leucine-rich proteoglycan (SLRP) being especially abundant within the reactive stroma that surrounds several human solid tumors^6, 7^. Since different and even opposite observations were done depending on both the cancer site as well as its molecular subtype^8^, it remains however unclear whether lumican should be seen as a putative prognostic indicator or whether its elevated expression is to be regarded as part of abundant stroma production within advanced tumors. Previous work by our group showed that decreased lumican expression in melanoma is associated with more infiltrative malignancies^9^. Consistently, *in vitro* studies revealed that lumican promotes melanoma cells adhesion^10^, while inhibiting their migratory capacities^11–13^. In addition, angiostatic properties were attributed to lumican in both normal^14^ and tumor^15^ vascular microenvironments. More recently, extracellular lumican was reported to enhance cytotoxicity of chemotherapy in multiple experimental models ranging from *in vitro* cell-based assays to transplantation of patient-derived xenografts^16^. All above-mentioned studies mostly focused on lumican ability to trigger signaling events in tumor cells and/or endothelial cells. In contrast, little attention has been given so far in studying lumican direct role in tumor matrix organization. Thus, it may be highly relevant to gain deeper insights about lumican-related modifications of matrix assembly that may impact tumor growth and/or dissemination. Indeed, multiple features of tumor ECM are likely to drive disease progression such as intratumoral pH, hydration, mechanical strengths but also diffusion of growth factors, stromal cells and therapeutic agents within a tumor mass. Breast cancer constitutes a blatant example for which alterations in ECM architecture have long-term been known as a prominent risk factor^17^, with both low lumican expression and more aligned collagen fibers correlating with poor outcome in this pathology^18, 19^.

In this report, a comprehensive review of available public clinical data for melanoma is first provided, highlighting a correlation between lumican expression and patient outcome. Using KO mice, we then extensively studied the role of host lumican on tumor ECM organization as well as on disease progression using an immunocompetent model of B16F1 melanoma allograft. To that end, a multimodal imaging approach was conducted combining histology, microvascular density (MVD), µCT angiography, tumor spectral imaging, but also polarization and second harmonic generation (SHG) microscopy. Innovative signal processing methodologies contributed to provide an accurate characterization of subtle changes that may occur within tumor ECM organization. Besides, we also sought to decipher whether endogenous lumican may modulate the response to ECM-targeted therapeutic strategy. Given the angiostatic properties that were previously attributed to lumican within a tumor microenvironment^15^, particular attention was paid to the influence of host lumican deficiency on both tumor vascularization and response to a matrix-targeted anti-angiogenic approach. To this end, an anticancer cyclic peptide that has previously been validated in the B16 allograft model^20^, named TAX2, was considered. TAX2 peptide targets matricellular thrombospondin-1 (TSP-1) at the CD47 binding site, therefore antagonizing TSP-1:CD47 interaction which is known to play a key role in both immune and angiogenic tumor responses^21, 22^. In both allograft and xenograft melanoma models, TAX2 peptide impacts tumor growth while sharply altering tumor-associated vascularization and decreasing intratumoral blood flow. In addition, TAX2 treatment also dramatically inhibits lung metastases dissemination and growth following invasive B16F10 melanoma cells tail vein inoculation^20, 23^. The proof-of-concept for TAX2 anti-angiogenic and anticancer properties was also confirmed using pancreatic as well as neuroblastoma tumor xenografts, in which systemic administrations at a 10 mg/kg body weight (BW) dose restricts tumor growth at least by 2-fold^20, 24^.

Overall, our study establishes for the first time a direct link between lumican expression and alterations in tumor ECM organization that support tumor growth in a melanoma preclinical model. Data obtained further indicate that such lumican-related structural changes are likely to sharply modulate tumor stromal reaction as well as response to matrix-targeting therapeutic strategies, as demonstrated considering TAX2 peptide treatment.

## Results

### Low lumican expression correlates with poor outcome in human melanoma

**Figure 1:**
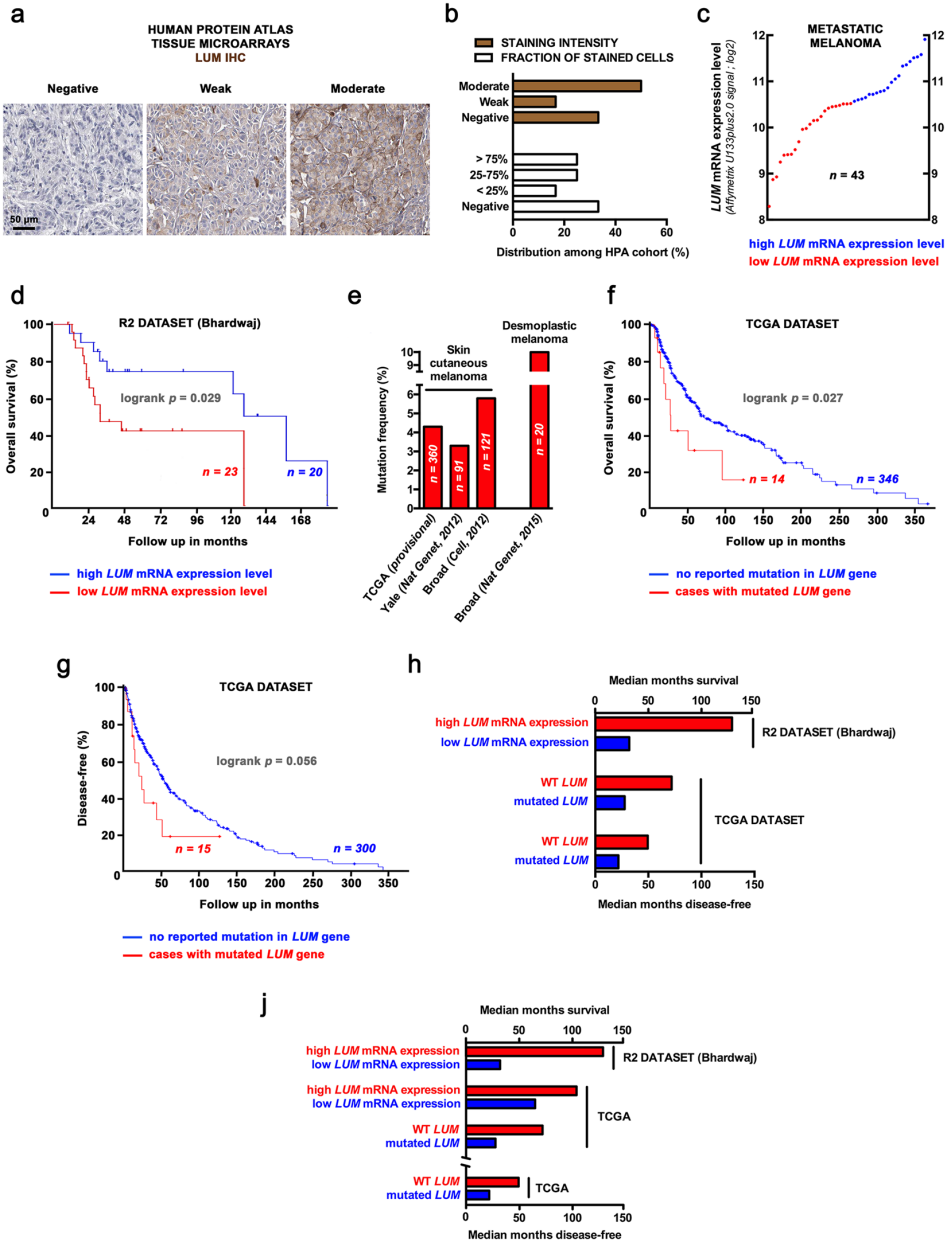
Correlation of lumican expression and outcome in melanoma patients: a comprehensive mining of available clinical databases. (**a**) Microphotographs of lumican (LUM) IHC in tissue microarrays from the Human Protein Atlas cohort (available from www.proteinatlas.org). Anti-LUM pAb (HPA001522, Sigma-Aldrich) has been used at a 1:150 dilution for IHC staining of tissues, followed by hematoxylin counter-coloration to enable visualization of microscopic features. (**b**) Distribution of melanoma cases among 3 levels of LUM staining intensity (*brown*) and fraction of cells stained for LUM expression (*white*). (**c**) Analysis of *LUM* mRNA expression levels (log2; Affymetrix U133plus2.0 signal) in microarray data for metastatic melanoma^26^. The log2 signal background for this probe (201744_s_at) is 6.37. *Red* and *blue circles* respectively stand for low and high *LUM* mRNA expression in 2 groups designed for further survival analysis. (**d**) Kaplan-Meier analysis for overall survival rates of 44 melanoma patients grouped by *LUM* mRNA expression. Logrank test raw *p* value is indicated. (**e**) Box-plot shows *LUM* expression across risk groups from TCGA dataset (SurvExpress tool), including the *p* value testing for difference using *t* test. Heat map displays *LUM* expression in risk groups, with low expression being represented in green grades and high expression in red grades. Calculated *β* coefficient from the Cox fitting (i.e. linear relationship between gene expression and prognostic index) was −0.121 for *LUM* gene. (**f**) Overall survival Kaplan-Meier plot for high (*blue*) and low (*red*) *LUM*-expressing risk groups is shown, with logrank *p* value being indicated. (**g**) Frequency of *LUM* somatic mutation among clinical samples from different cohorts. Data were obtained from the cBioPortal for cancer genomics. (**h**) Overall and (**i**) disease-free survival curves of TCGA patients presenting skin cutaneous melanoma with (*red*) or without (*blue*) *LUM* mutations. (**j**) Histogram summarizes median overall survival values (months) in above-mentioned molecular subgroups across R2 (*top*) and TCGA (*middle*) datasets as well as median disease-free survival among TCGA patients (*bottom*). GEO, Gene Expression Omnibus; TCGA, The Cancer Genome Atlas.

Lumican was previously reported as being expressed within tumor stroma of malignant melanoma, while inversely correlating with malignancy according to Clark levels that reflect disease vertical progression^9^. Consistently, the pattern of lumican immunohistochemistry (IHC) staining among the Human Protein Atlas cohort^25^ shows differential protein expression ranging from total absence of lumican within dense tumor tissue to a moderate staining of both cancer and stromal cells (Fig. 1a and b). Analysis of lumican-encoding gene (*LUM*) expression among 44 cases of metastatic melanoma^26^ confirmed such heterogeneity (Fig. 1c). Among this cohort, *LUM* gene expression correlates with survival in patients with melanoma, with a 4-fold increase in median overall survival being observed in the high lumican-expressing group, as determined using the optimal cut-off (calculated using the R2 web tool) of *LUM* mRNA expression (Bhardwaj dataset, GEO accession number GSE19234, Fig. 1d). Study was then extended to include data from The Cancer Genome Atlas (TCGA), among which separation was performed between risk groups characterized by differences in *LUM* gene expression. We found that *LUM* expression levels significantly correlate with a lower risk of poor prognosis for skin cutaneous melanoma patients (Fig. 1e), and therefore confirmed that overall survival is longer in cases exhibiting higher *LUM* expression (Fig. 1f). Interestingly, alterations in *LUM* gene sequence are found in about 5% of clinical samples in TCGA dataset as well as across 3 independent studies (Fig. 1g), with highest mutation rates being observed in desmoplastic melanoma i.e. a deeply infiltrating subtype of melanoma with abundant stroma^27^. A decrease in both overall and disease-free survival of TCGA patients is observed when mutations in *LUM* gene sequence are reported (Fig. 1h and i), thus correlating with above-mentioned mRNA expression studies (Fig. 1d,f and j). Altogether, this first round of data arising from multiple databases analyses undoubtedly establishes a relationship between altered lumican expression and poor clinical outcome in human melanoma.

### Endogenous lumican inhibits tumor growth and modulates response to ECM-targeting TAX2 peptide in a melanoma allograft model

**Figure 2:**
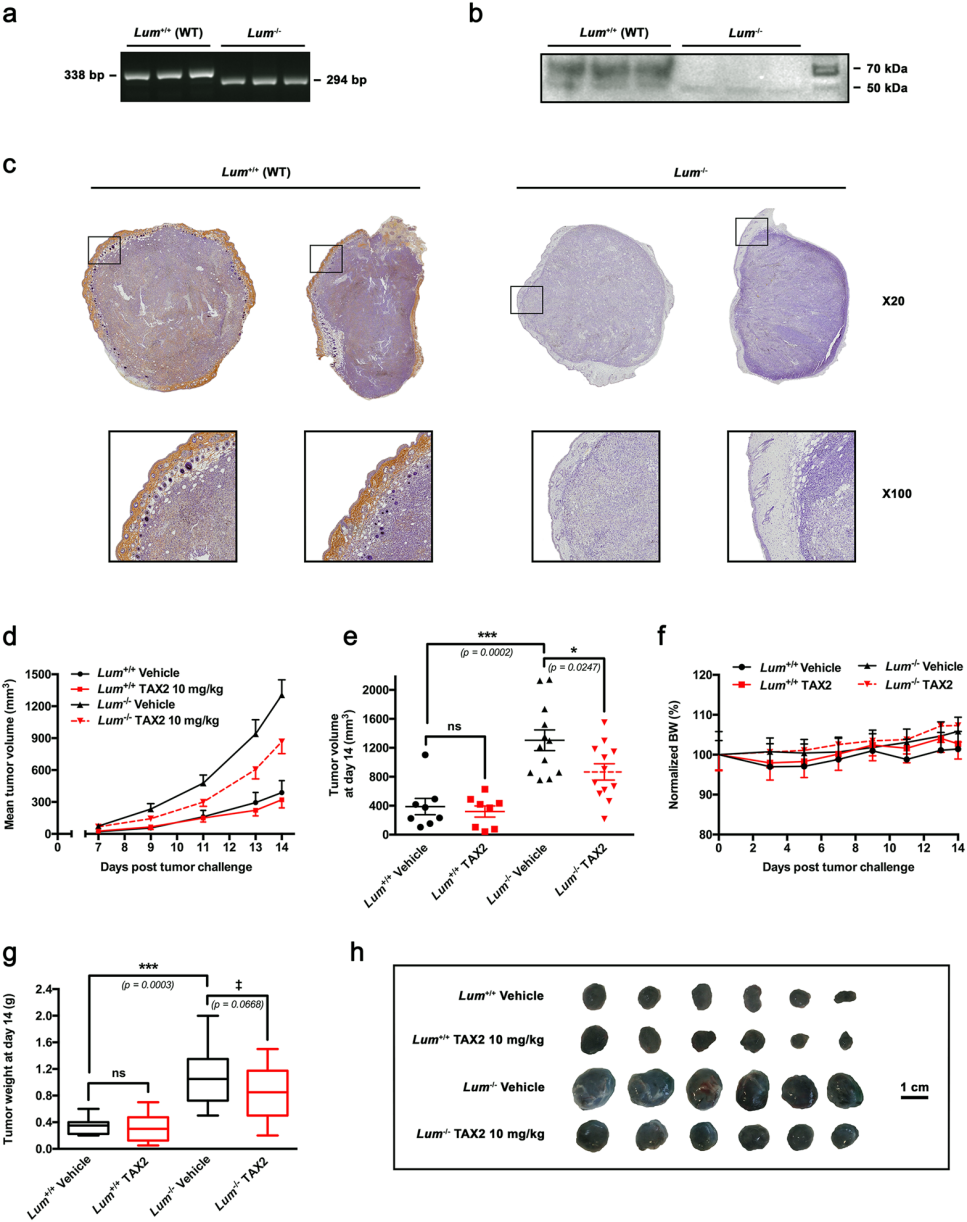
Evaluation of endogenous lumican impact on tumor growth and response to TAX2 treatment in a melanoma allograft model. (**a**) Genotype of representative offsprings from wild type (*Lum* ^+/+^) matings and lumican deficient (*Lum* ^−/−^) matings by PCR analysis. (**b**) Western immunoblotting on total protein extracted from skin of wild type (*Lum* ^+/+^) mice (*n* = 3) and lumican-deficient (*Lum* ^−/−^) mice (*n* = 3) with a rabbit polyclonal antibody raised against lumican core protein. Protein extracts from wild type mice exhibit a broad band at 57–90 kDa indicating the presence of glycosylated lumican as expected. Skin from lumican-deficient (*Lum* ^−/−^) animals lacks any immunoreactive material confirming the absence of any lumican gene product. (**c**–**h**) B16F1 melanoma cells (2.5 × 10^5^) were s.c. inoculated in *Lum* ^+/+^ or *Lum* ^−/−^ syngeneic C57BL/6 J mice, and then i.p. administrations of either Vehicle (0.9% NaCl) or TAX2 peptide (10 mg/kg) were performed on days 3, 5 and 7 post tumor cells inoculation. On day 7, tumors were detectable and tumor volume was measured every 1–2 days as described in Materials and Methods. (**c**) Microscopic views of s.c. allograft whole sections IHC (*top panel*, ×20) allowing visualization of lumican within tumors implanted in wild-type (*Lum* ^+/+^) mice while it is neither detected in tumors from *Lum* ^−/−^ animals. *Insets* show higher magnification (×100) of stromal margin surrounding melanoma allografts. (**d**) Averages of calculated tumor volumes in mm^3^ (mean ± SEM, *n* = 8–12 per group). (**e**) Scatter dot plot of individual calculated tumor volumes on day 14. *Line*, mean ± SEM (*t* test, ns not significant, *p < 0.05, ***p < 0.001). (**f**) Evolution of normalized mice body weights (BW), expressed as a percentage of day 0 (mean ± SEM). (**g**) Tukey’s box and whisker plots of isolated tumor weight in g. Significant (***p < 0.001) as well as marginally significant (^‡^ *p* < 0.10) differences between groups are indicated (*t* test). (**h**) Representative photographs of B16F1 melanoma s.c. allografts after tumor excision.

Earlier *in vivo* work using B16F1 cells engineered to express lumican strongly suggested its involvement in the control of melanoma progression^15^. However, such approach mainly depending on lumican ectopic expression from inoculated cancer cells is not relevant in understanding the contribution of host lumican. Using knockout mice (*Lum* ^−/−^), the present report demonstrates for the first time that lumican is an endogenous inhibitor of melanoma tumor growth. Lumican deficiency was first ensured by PCR genotyping, therefore clearly showing that the size of the amplicon is shorter in lumican-deleted mice than in wild type mice, as expected (Fig. 2a). In addition, western immunoblotting on total protein extracted from skin of wild type mice shows a broad band at 57–90 kDa indicating the presence of glycosylated lumican, while skin from *Lum* ^−/−^ mice lacks any immunoreactive material, confirming the absence of any lumican gene product (Fig. 2b). IHC analysis of lumican reveals a strong staining in the dermis and especially at the stromal margin surrounding melanoma allografts implanted in WT mice (Fig. 2c, *left panel*). Additionally, a weak staining could be observed between melanoma cells, while lumican was not detected in the epidermis as expected. In contrast, lumican was neither detected in the dermis nor in stromal cells within tumor from *Lum* ^−/−^ animals, therefore confirming the lumican-null phenotype (Fig. 2c, *right panel*). Strikingly, syngeneic B16F1 allograft growth was more than three times increased in *Lum* ^−/−^ mice as compared to their WT (*Lum* ^+/+^) littermates (Fig. 2d). So as to decipher whether host lumican deficiency may modulate tumor response to ECM-targeted therapeutic approach, we considered a previously validated anti-angiogenic peptide named TAX2^20, 22–24^. Interestingly, TAX2 peptide induces a 40% reduction in tumor burden from lumican-deficient mice (Fig. 2d and e) while it does not affect allograft volume in WT mice, as previously described^20^. No treatment-related toxicity was reported as no adverse clinical signs nor BW loss (Fig. 2f) nor mortality/morbidity were detected. Weighting of surgically-removed allografts confirmed longitudinal tumor volume calculations (Fig. 2g). Indeed, tumors recovered from *Lum* ^−/−^ mice were approximately two-fold larger respectively than tumors from WT mice, while TAX2 treatment induces a 30% diminution in tumor size only in *Lum* ^−/−^ mice (Fig. 2h).

### Lumican deficiency impacts intratumoral distribution of matricellular proteins, growth factors and stromal cells

**Figure 3:**
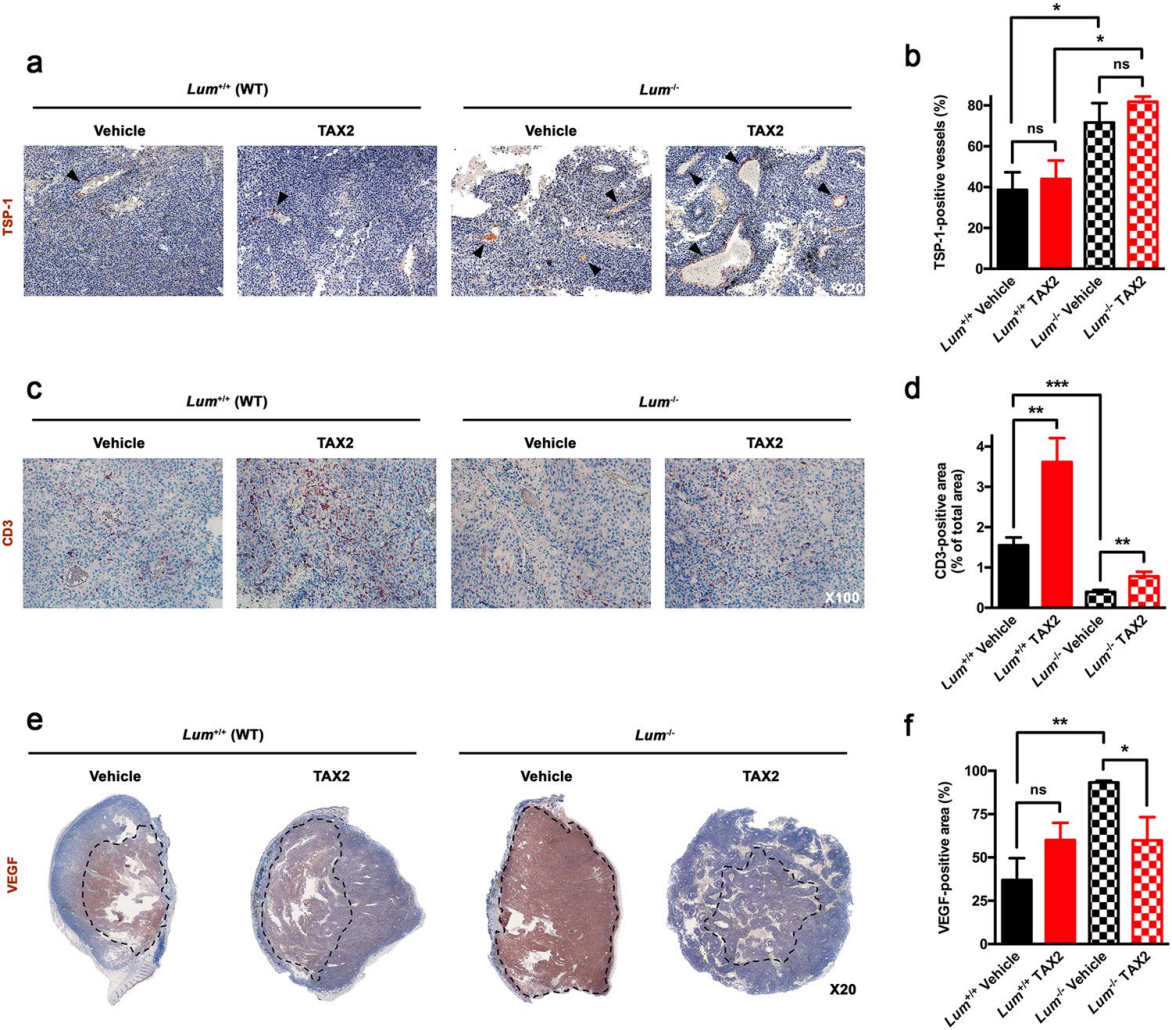
IHC analyses of s.c. melanoma allografts stromal features. (**a**–**f**) Microscopic views of tissue sections IHC allowing visualization of intratumoral (**a**) TSP-1 (×20, *black arrowheads*), (**c**) CD3 (×100) and (**e**) VEGF (×20, *dotted lines*) immunostainings and quantification of (**b**) percentage of TSP-1-positive blood vessels as well as relative (**d**) CD3-positive and (**f**) VEGF-positive areas (mean ± SEM, *t* test, ns not significant, *p < 0.05, **p < 0.01, ***p < 0.001). All quantitative analyses were performed using ImageJ software. Five random fields were considered for TSP-1 and CD3 immunostainings, while the whole tumor section was analyzed for VEGF positivity.

As TAX2 peptide was designed so as to specifically bind the carboxy-terminal domain of matricellular TSP-1^20^, its levels within s.c. melanoma allografts were first investigated through IHC analyses. Higher rates of TSP-1 were found within tumors engrafted to *Lum* ^−/−^ mice as compared to WT mice (Fig. 3a and b), especially accumulating at sites of intratumoral macrovessels (Fig. 3a, see *black arrowheads*) and around necrotic as well as hemorrhagic areas. TSP-1 binding to CD47 cell-surface receptor was previously demonstrated to inhibit antitumor adaptative immunosurveillance^22, 28, 29^. Indeed, TAX2-mediated TSP-1:CD47 antagonization led to a two-fold increase in CD3^+^ T cell infiltration in treated tumors from both WT and *Lum* ^−/−^ mice (Fig. 3c and d). Interestingly, we also noticed a 75% decrease in basal levels of tumor-infiltrating T cells under lumican deficiency. Besides, exposition to the pro-angiogenic vascular endothelial growth factor (VEGF) was found to be enhanced within tumors from lumican-deficient animals, while being downregulated under TAX2 treatment in *Lum* ^−/−^ animals but not in WT ones (Fig. 3e and f).

### Lumican host expression modulates tumor vascularization and response to anti-angiogenic TAX2 treatment

**Figure 4:**
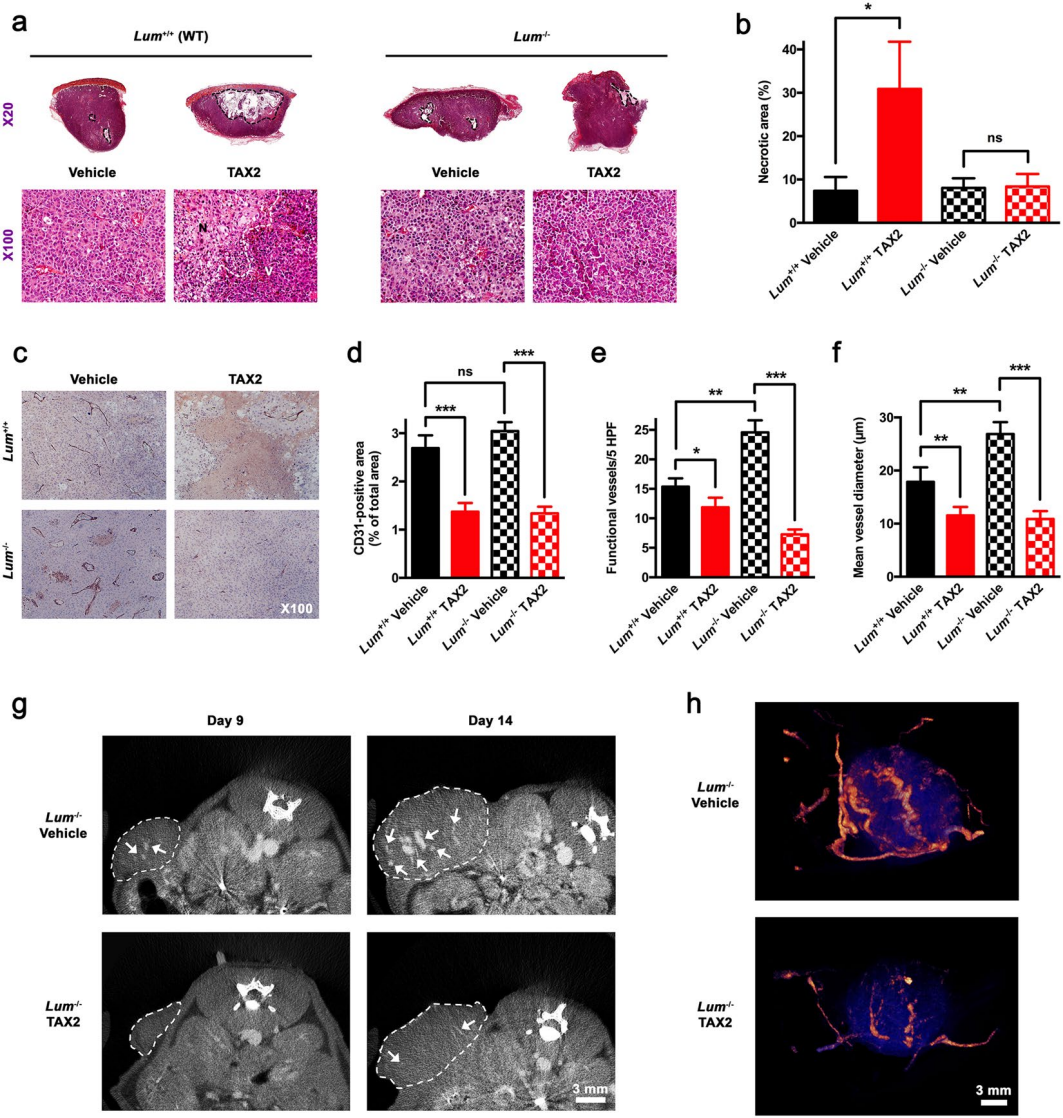
Analysis of s.c. melanoma tumor necrosis and vascularization. (**a**) Macroscopic views (x20 magnification, *top panel*) of HES-stained sections showing areas of tumor necrosis (*black dotted lines*). *Lower panel* (×100 zoom) highlights delimitation (*white dotted line*) between viable tumor tissue (V) and cellular debris within necrotic area (N). (**b**) Quantification of tumor necrosis relative to total tumor surface (mean ± SEM, *t* test, ns not significant, *p < 0.05). (**c**) CD31 immunostainings of tumor sections (×100). (**d**–**f**) Quantification of (**d**) relative CD31-positive area, (**e**) number of functional blood vessels in five high power fields (HPF, ×100) and (**f**) mean vessel diameter (mean ± SEM, *t* test, ns not significant, *p < 0.05, **p < 0.01, ***p < 0.001). All quantitative analyses were performed using ImageJ software and considering five random fields per animal. (**g**) Longitudinal follow-up of tumor angiography through µCT analysis. *Dotted lines* delimitate s.c. tumors while *arrows* illustrate contrast enhancement of intratumoral blood vessels. (**h**) Tridimensional reconstructions of tumors imaged on day 14. Melanoma tumor (*blue*) and the associated vascular network (*orange*) were segmented using Amira 5.4.3 software.

Morphologic analyses of B16F1 s.c. tumors confirmed earlier work that demonstrated a pro-necrotic effect of TAX2 peptide administration in tumor-bearing WT animals^20^, while no TAX2-related necrosis was observed in tumors from *Lum* ^−/−^ mice (Fig. 4a and b). Yet, IHC observations as well as quantitative analysis of CD31-positive areas highlighted a potent anti-angiogenic effect associated to TAX2 treatment in both WT and *Lum* ^−/−^ mice (Fig. 4c and d). Consistent with prior reports suggesting a putative anti-angiogenic activity of lumican under certain conditions^30–32^, results arising from the B16 melanoma allograft model highlighted that the number of intratumoral functional vessels as well as their mean diameter are indeed increased under lumican deficiency (Fig. 4e and f). Interestingly, TAX2 inhibitory effects regarding these vascularization parameters appeared even larger in *Lum* ^−/−^ mice as compared to those observed in WT mice, therefore demonstrating an influence of lumican host expression on tumor inhibitory anti-angiogenic response. At last, results of immunohistological analyses were corroborated by micro-computed tomography (µCT) longitudinal follow-up of tumor-associated vascular network, supporting strong anti-tumor and angiostatic properties of TAX2 peptide in *Lum* ^−/−^ mice (Fig. 4g and h).

### Lumican host expression drives biochemical heterogeneity of s.c. implanted melanoma tumors

**Figure 5:**
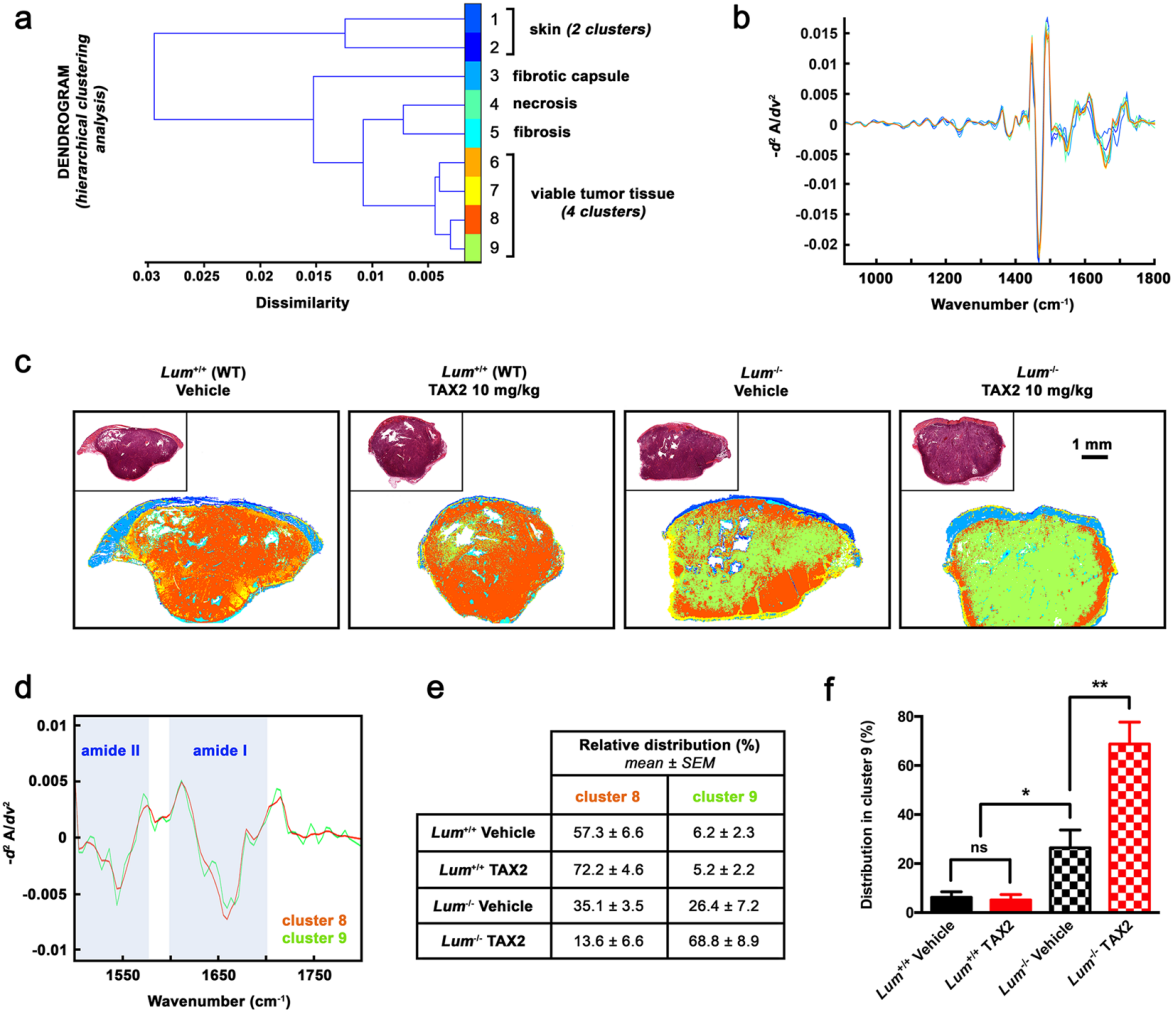
*K*-means clustering of tumor FT-IR spectral images. (**a**) Dendrogram obtained as a result of hierarchical clustering showing spectral dissimilarity between the 9 cluster centroids estimated by unsupervised *K*-means clustering of s.c. tumor infrared images. Random pseudo-colors were attributed to each cluster, while comparison to adjacent HES-stained sections allowed histological annotations of *K*-means subclasses. (**b**) Second derivative spectra (900–1800 cm^−1^) of corresponding cluster centroids. (**c**) Representative color-coded *K*-means clustered images of tumor sections. All eliminated spectra by extended multiplicative signal correction algorithm (i.e. pure paraffin and low signal-to-noise ratio spectra) were colored as white pixels. *Insets* show adjacent HES-stained tissue sections. (**d**) Second derivative spectra (1500–1800 cm^−1^) of centroids of clusters 8 (*orange*) and 9 (*green*). Most discriminant amide II and amide I bands are shown as *blue bars*. (**e**) Mean relative distribution of pixels attributed to clusters 8 and 9 among tumors from animal subgroups, expressed as a percentage of total pixels. (**f**) Histogram shows results from statistical analysis of relative distribution within cluster 9 (mean ± SEM, *t* test, ns not significant, *p < 0.05, **p < 0.01).

In order to assess subtle changes that may occur in tumor molecular assembly depending on whether host animals do express lumican or not, an innovative method relying on unsupervised *K*-means clustering of FT-IR spectral images was then used^33, 34^. Second derivative spectra obtained from s.c. melanoma allografts were partitioned into 9 clusters being representative of tissue biochemical properties^34, 35^ (Fig. 5a and b). Resultant pseudo-color images highlight an increased heterogeneity within tumors from *Lum* ^−/−^ mice, characterized by dissimilar distribution of *K*-means subclasses that correspond to viable tumor tissue (Fig. 5a and c). Of note, the emergence of a cluster being specific for tumors implanted in lumican-deficient animals was further amplified under TAX2 treatment (Fig. 4c, *right panel*). Among intrinsic spectral features of clusters 8 and 9 identified as the most discriminant between tumors from *Lum* ^−/−^ and WT mice (Fig. 5a and c), differences were notably observed within amide I and amide II bands that are known as being predictive for protein structure^36^ (Fig. 5d). Analyses of relative pixel distribution further characterized the above-mentioned TAX2-related striking effect, which can only be observed in *Lum* ^−/−^ mice that display increased tumor tissue heterogeneity (Fig. 5e and f). Altogether, this FT-IR microspectroscopy approach coupled to unsupervised clustering allowed detection of slight structural differences within tumor viable tissue, that were not viewable through conventional histology. Particularly, the co-existence of 2 predominant clusters within viable tissue from tumors implanted in *Lum* ^−/−^ mice constitutes the first evidence for increased intra-tumor heterogeneity induced by the lumican-null phenotype. Such decrease in tumor structuration level sharply suggests an altered matrix content, therefore we thought to further analyze intratumoral collagen deposition as well as organization within s.c. allografts.

### Endogenous lumican deficiency decreases intratumoral collagen deposition

**Figure 6:**
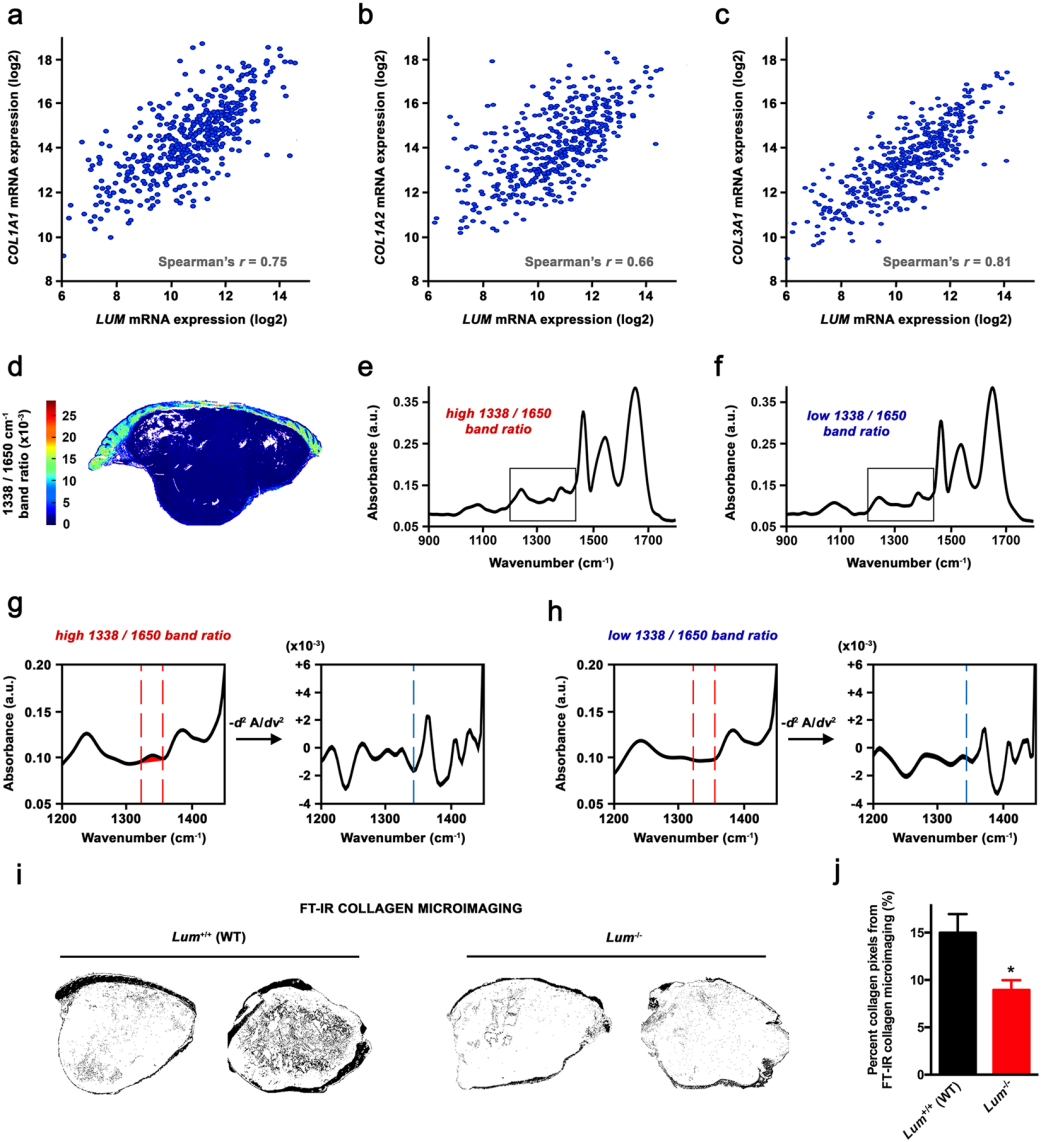
Investigation of the relationship between lumican and collagen deposition within tumor. (**a**–**c**) Levels of mRNA (z-scores) for *COL1A1* (**a**), *COL1A2* (**b**) and *COL3A1* (**c**) genes are plotted against *LUM* mRNA levels in clinical samples of 473 skin cutaneous melanoma cases from the TCGA dataset. Indicated *r* values were calculated using Spearman’s rank correlation test. (**d**–**j**) FT-IR microimaging of collagen in s.c. melanoma allografts implanted in *Lum* ^+/+^ *vs*. *Lum* ^−/−^ mice. (**d**) Image obtained by integration on absorbance band at 1338 cm^−1^ arising from collagen sidechain vibrations. Color bar indicates corresponding calculated 1338/1650 cm^−1^ ratio. (**e**–**h**) Representative spectra for high (**e**) and low (**f**) 1338/1650 cm^−1^ ratio. *Insets* focus on the 1200–1450 cm^−1^ spectral range that is displayed in (**g**) and (**h**), showing the area of integration for 1338 cm^−1^ absorbance in *left panels*. *Right panels* display corresponding second derivative spectra so as to facilitate peak identification (*blue dotted line*). (**i**) Representative binary images separating collagen from the rest of the tumor tissue after FT-IR microimaging of collagen deposition (1338 cm^−1^ area). (**j**) Quantification of the number of collagen pixels relative to total tumor pixels (%) implanted in *Lum* ^+/+^ (WT) *vs*. *Lum* ^−/−^ mice (mean ± SEM, *t* test, *p < 0.05), as performed using ImageJ software.

Lumican has long time been recognized as a regulator of collagenous matrix self-assembly^37, 38^. Hence, we sought to determine whether the impact of lumican on tumor ECM topology may depend on collagen fibrillogenesis. Co-expression analyses were first performed among TCGA melanoma dataset, highlighting that human *LUM* mRNA levels correlate with expression of genes encoding for α chains of type I (Fig. 6a and b) and type III (Fig. 6c) collagens, i.e. two of the most abundant matrix components within a tumor microenvironment^39^. Then, a Fourier transform infrared (FT-IR) imaging spectroscopy methodology was used for detection and quantitative analyses of collagen deposition within B16F1 s.c. allografts. This alternative approach overcomes the variability experienced in conventional staining techniques^40^ and allows high resolution and automated data collection without addition of any chemical or histology reagent to the tissue sample. Briefly, the 1338 cm^−1^ IR absorbance band arising from collagen amino acid side chain vibrations was used to map collagen deposition across tumor tissue (Fig. 6d to h). The results obtained highlighted a 40% decrease in the collagen content of tumors from *Lum* ^−/−^ mice as compared to WT (Fig. 6i and j), thus sustaining a role for collagen in lumican-dependent regulation of tumor ECM assembly.

### Endogenous lumican deficiency impairs intratumoral collagen organization

**Figure 7:**
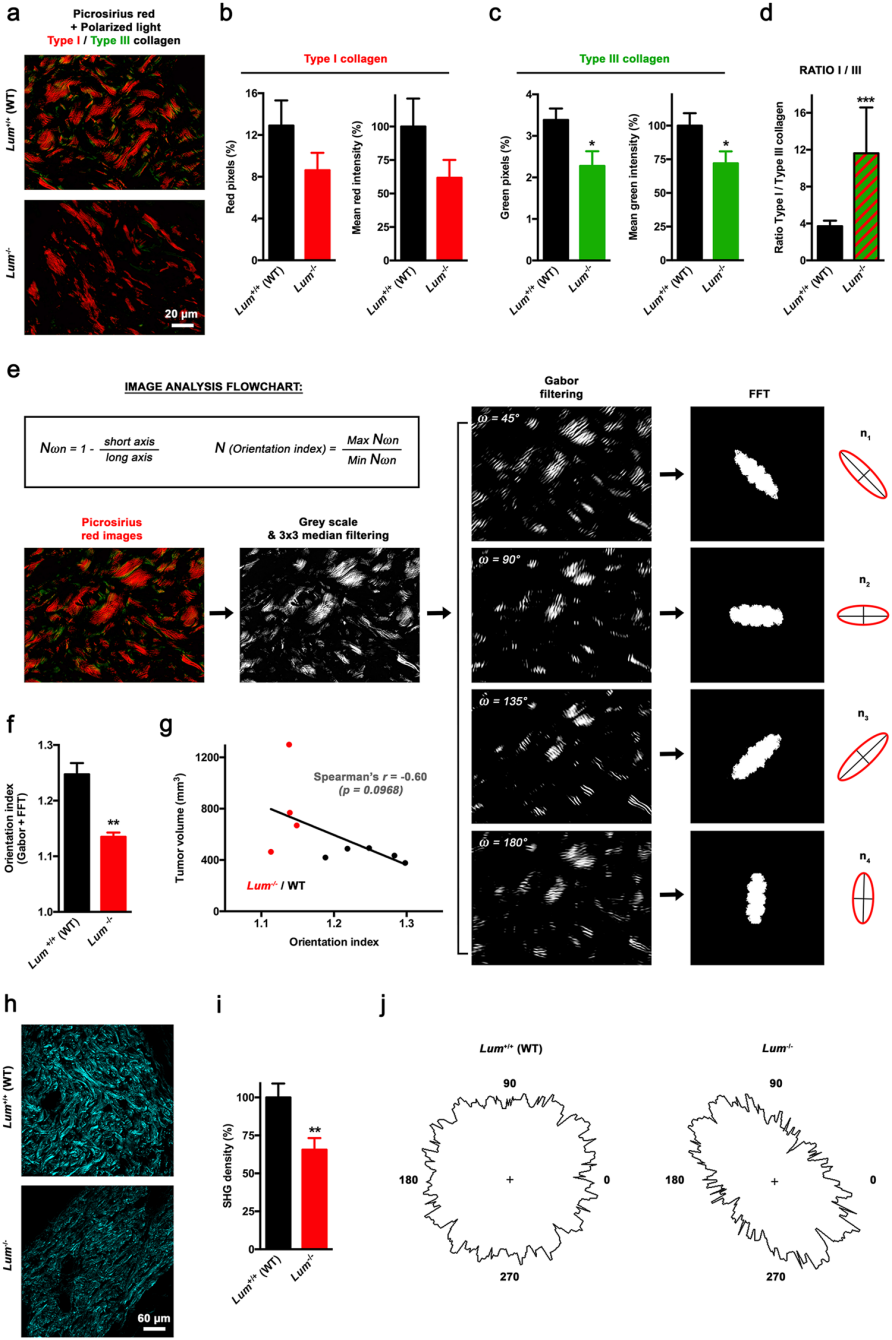
Investigation of the relationship between lumican and intratumoral collagen organization. (**a**) Representative microphotographs of s.c. allograft sections stained with picrosirius red and viewed under dark-field cross-polar optics (original magnification ×63). Birefringence of collagen fibers allows distinction between type I (red) and type III (green) collagen. (**b**,**c**) Quantification of the relative distribution of red (**b**, *left panel*) and green (**c**, *left panel*) pixels as well as the corresponding intensities (**b** and **c**, *right panels*), expressed as a percentage of mean pixel intensity in the *Lum* ^+/+^ (WT) group (mean ± SEM, *t* test, *p < 0.05). (**d**) Quantification of type I/type III ratio based on red and green pixel calculations, respectively. Individual ratios were calculated from 3 different fields per animal, and then averaged (mean ± SEM, *t* test, ***p < 0.001). (**e**–**g**) Quantification of tumor ECM collagen organization from spectra derived from Gabor filtering and FFT. (**e**) Flowchart depicting the different stages of image analysis. From grey scale-converted picrosirius-stained images, a 3 × 3 median filter is applied before Gabor filtering and then FFT. After determining the elliptical shape of the scatter pattern (see *red* ellipses on *right panel*), measurements of the elliptical axes (*black lines*) generated from ω angle values n_1_ to n_4_ were performed so as to produce a collagen orientation index (N), according to displayed equations (*inset*). (**f**) Histogram displays results of collagen orientation index determination within melanoma tumors allografted to *Lum* ^+/+^ or *Lum* ^−/−^ mice (mean ± SEM, *t* test, **p < 0.01). (**g**) Correlation between calculated collagen orientation index and final tumor volume in *Lum* ^+/+^ (WT, *black dots*) and *Lum* ^−/−^ (*red dots*) mice. Linear regression was performed (*black line*) and *r* coefficient arising from non-parametric two-tailed Spearman test was determined. (**h**) Representative collagen SHG images from B16F1 tumors (original magnification ×20). (**i**) Collagen density of SHG images for s.c. allografts of *Lum* ^+/+^ and *Lum* ^−/−^ animals (mean ± SEM, *t* test, **p < 0.01). (**j**) Representative polar plots of SHG intensity *vs*. angle of laser polarization.

To go further ahead in the characterization of intratumoral collagen organization, s.c. allografts sections were then visualized under polarized light following picrosirius red staining (Fig. 7a). Pixel counts as well as mean intensity calculations highlighted a 30–40% decrease in signals arising from both type I (Fig. 7b) and type III (Fig. 7c) collagen in *Lum* ^−/−^ mice as compared to WT. Conjointly, individual type I/type III ratios were also significantly affected under lumican deficiency (Fig. 7d). An original analytical approach combining Gabor filtering and Fast Fourier Transform (FFT) before spectra analysis was then used to allow measurement of collagen basketweave structure integrity, including detection of slight changes that remains intractable from other existing techniques^41^ (Fig. 7e). Orientation indexes computation emphasized a loss of collagen organization within tumors implanted in *Lum* ^−/−^ mice (Fig. 7f), which correlates with linearized collagen fibers observed microscopically (Fig. 7a). Although not significant (*p* = 0.09), there was a clear trend towards a correlation between calculated orientation index and tumor volume at study termination (Fig. 7g). Finally, second harmonic generation (SHG) imaging of s.c. allografts (Fig. 7h) corroborates above-mentioned findings, as both decreased SHG density (Fig. 7i) and unidirectional-oriented fibers (Fig. 7j) were observed in absence of host lumican expression.

## Discussion

A comprehensive and large-scale mining of public microarray databases was first performed in this study, thus unambiguously demonstrating a correlation between altered lumican expression and poor outcome in human melanoma. Then, B16 melanoma allografts were transplanted in both WT and *Lum* KO mice in order to characterize the influence of host lumican on tumorigenesis. Taken together, the results obtained from this model indicate that (*i*) lumican acts as a potent endogenous inhibitor of tumor growth, while (*ii*) newly establishing lumican as a key driver of tumor matrix assembly. At the same time, a previously validated anticancer peptide targeting matricellular TSP-1^20, 22–24^ was considered so as to figure out lumican-related modulation of tumor response to ECM-targeted therapeutic strategies. Interestingly, our data show that lumican expression within host tissues affects both soluble factors as well as immune and endothelial cells intratumoral distribution, hence sharply modifying tumor response to TAX2 peptide.

Bioinformatics analyses of both mRNA and protein levels of lumican among melanoma clinical cases highlighted much heterogeneity (Fig. 1a to c), which is consistent with discrepancies that previously arose when looking at *in vitro* lumican expression depending on the cell line used^9, 42^. A thorough screening of multiple melanoma clinical datasets further highlighted that both alterations in *LUM* gene sequence as well as lower mRNA expression are associated with decreased patient survival (Fig. 1d to j). This should be put in perspective with previous work underlying decreased lumican expression within the most infiltrative melanoma lesions^9^. Besides, computation of genome-wide expression among TCGA revealed that *LUM* mRNA levels correlate with genes encoding for the α chains of type I (i.e. *COL1A1* and *COL1A2*) and type III (i.e. *COL3A1*) collagens (Fig. 6a to c). Such intriguing finding was further corroborated through FT-IR mapping of collagen within s.c. B16 allografts that highlighted a decrease in intratumoral collagen deposition under lumican deficiency (Fig. 6i and j). Supporting these *in vivo* observations, we previously evidenced that lumican inhibits the collagenolytic activity of matrix metalloproteinase 14 (MMP14) in B16F1 cells^43^. Contrary to what is observed regarding collagen-encoding genes (Fig. 6a to c), our present bioinformatics studies further revealed that *MMP14* inversely correlates with *LUM* expression in melanoma, with decreased survival being related to higher *MMP14* expression (Suppl Fig. S1). This finding keeps in line with results from other groups that earlier reported lumican interaction at the surface of collagen fibrils as a way to protect against cleavage by other collagenases^44^. Interestingly, membrane-bound MMP14 (MT1-MMP) appears as a particularly relevant target within lumican interactome, as it is especially well known to be a positive regulator of angiogenesis in collagen-rich environments^45^. Indeed, MMP14-related control of ECM remodeling^46^ as well as VEGF expression and bioavailability^47, 48^ provides hypothetical explanations of our present results obtained from lumican-deficient mice, and thus regarding both melanoma allografts vascularization (Fig. 4c to f) as well as VEGF distribution within tumors (Fig. 3e and f).

Our data clearly indicate that lumican deficiency is associated with fast-growing melanoma allografts (Fig. 2d) that display increased biochemical heterogeneity, as revealed using unsupervised *K*-means classification of tumor tissue FT-IR absorbance spectra (Fig. 5). Tumors from *Lum* ^−/−^ mice also exhibit a lower collagen content as compared to those engrafted in WT animals (Fig. 6i and j). Interestingly, such decrease in collagen deposition is likely to be tumor-specific as either polarized or SHG microscopy highlighted no difference in collagen density within normal skin of *Lum* KO *vs*. WT animals (Suppl Fig. S2). Nevertheless, our data singularly contrast with extensive literature that acknowledges increased ECM deposition as a hallmark of tumor progression^49–51^. To explain this apparent discrepancy, one can rely on previous work highlighting that re-orientation of collagen fibers within a tumor microenvironment is central to the progression of cancer, being even more important than changes in collagen network amount and stiffness^2^. Based on original image analysis methodologies, the first experimental evidence that lumican effectively plays a major role in tumor ECM organization is provided in the present report. Indeed, a striking linearization of interstitial collagens is observed within tumors recovered from lumican-deficient mice as compared to WT (Fig. 7f and j), with those typical morphological changes being correlated to a promotion of tumor growth (Fig. 7g). Based on available clinical data, breast cancer is a typical example supporting that collagen alignment may be considered as a poor prognosis indicator in the clinics^52^. Besides, it has recently been shown using tridimensional cell culture models that linearized collagen promotes the acquisition of an invasive phenotype by mammary tumor cells, while high-density non-fibrillar collagen does not^53^. These conclusions further sustain the concept that collagen structuration rather than density alone modulates cancer cell behavior. Interestingly, low lumican expression has early been related to larger tumor size, recurrence and poorer survival in patients presenting invasive breast carcinoma^18^. Short while ago, several proteomics analyses additionally identified alterations in lumican expression in various cancer types including osteosarcoma^54^ as well as renal^55^ and ovarian^56^ cancers. In light of these findings, our approach may be particularly relevant in studying the role of extracellular lumican in the aforesaid tumor contexts. Indeed, further work will be aimed to decipher its influence on disease progression while considering highly metastatic tumors with abundant stroma such as ovarian carcinoma.

ECM re-organization and modifications in collagen structure, especially at tumor invasion front, are now well recognized to promote metastatic dissemination along aligned fibers^39, 57^. Therefore, identifying new specific molecular targets as well as providing a deeper characterization of stages of tumor collagen assembly present a great therapeutic challenge^58^. For instance, a recent study considering a mouse model a breast cancer xenografts demonstrated the benefit of disrupting lysyl oxidase like 2 (LOXL2)-mediated ECM fibril alignment^59^. Beyond its passive structural role, ECM remodeling also widely impacts tumor biology, including growth factor and/or membrane receptors distribution as well as stromal cells infiltration. As way of example, collagen linearization has been shown to promote redistribution of β1-integrin away from the plasma membrane of mammary tumor cells, which is consistent with the above-mentioned acquisition of an invasive phenotype in 3D models^53^. In view of these results, it cannot be ruled out that modifications in collagen network architecture may affect subcellular distribution of a wider range of cell receptors, hence sharply modifying communications that establish at the cell/matrix interface as well as tumor response to ECM-targeted strategies. As well, disorganization of tumor collagen network may also affect soluble factors and matricellular proteins intratumoral distribution. This is likely to affect several biological features of tumors, including blood vessels formation. Hence, we found that host lumican deficiency promotes a pro-angiogenic tumor phenotype through significant increase of both intratumoral functional vessels number (Fig. 4e) and diameter (Fig. 4f). Interestingly, CD31 immunostainings and µCT analysis of tumor angiography highlighted that the angiostatic properties of TAX2 peptide targeting TSP-1:CD47 interaction are preserved in such a pro-vascular microenvironment. Indeed, some of TAX2-mediated anti-angiogenic effects appeared even more pronounced in *Lum* KO *vs*. WT mice (Fig. 4e and f), which is consistent with enhanced bioavailability of both TAX2 direct (i.e. TSP-1, Fig. 3a and b) and indirect (i.e. VEGF, Fig. 3e and f) molecular targets under lumican deficiency. By contrast, TAX2 treatment failed to induce any tumor necrosis in *Lum* KO mice (Fig. 4a and b), while it leads to a typical pro-necrotic tissue response within tumors implanted in WT mice^20^. This latter observation strongly suggests that TAX2-related antitumor effects are indeed not only dependent on tumor angiogenesis, but may involve other mechanisms.

Apart from its role in modulating tumor angiogenic response, TSP-1/CD47 signaling also leads to tolerogenic signals that allow tumor immune escape, through direct inhibition of T cell activation^29, 60^ as well as by regulating natural killers and dendritic cells functions^61, 62^. Disruption of TSP-1:CD47 interaction under TAX2 treatment indeed promotes accumulation of infiltrating CD3+ T cells within melanoma tumors implanted in WT mice (Fig. 3c and d), especially around vascular structures and next to necrotic areas (data not shown). Such TAX2-mediated two-fold increase in CD3-positive area was also observed in *Lum* KO mice (Fig. 3d), therefore further confirming the immunomodulatory potential of TAX2 peptide in this immunocompetent tumor model. Yet, it has to be noticed that the baseline level of tumor-infiltrating T lymphocytes surprisingly shows a marked 75% decrease under lumican deficiency (Fig. 3d). This observation is nevertheless consistent with other studies supporting a role for lumican in inflammatory processes and immunoresponse in mouse non-cancer models such as corneal healing^63^ or colitis^64^. Interestingly, the decrease in T cell infiltration that is observed within tumors from *Lum* KO mice (Fig. 3c and d) might also be related to the concomitant above-mentioned increase in VEGF bioavailability (Fig. 3e and f), as VEGF was previously shown to carry immunosuppressive activity in T cells^65^. To our knowledge, the present study is the first to report a role for lumican in sustaining adaptive antitumor immunity. Therefore, ongoing studies are now aimed at further deciphering the pathway involved, as well as to investigate a putative role for lumican in fostering tumor response to immunotherapeutic approaches such as immune checkpoint inhibitors anti-PD-1 and anti-CTLA-4.

Overall, our findings based on innovative and multimodal approaches constitute, to our knowledge, the first demonstration for the key role of extracellular lumican in organizing tumor molecular assembly. Based on prior knowledge regarding the use of a thrombospondin-targeting anticancer peptide in the same melanoma allograft model^20^, we further highlighted that lumican host expression strongly influences tumor response to ECM-targeted therapy. Together, data obtained provide new insights in defining lumican as a diagnostic marker and/or therapy target. Besides, this will also guide future directions in TAX2 peptide preclinical development, both regarding a preferred utilization in highly stromal tumors as well as its positioning as an add-on to current immunotherapeutic approaches.

## Materials and Methods

### Bioinformatics and clinical data mining

The pattern of LUM staining in melanoma clinical samples was characterized using the Human Protein Atlas web portal (available from www.proteinatlas.org)^25^. Semi-quantitative analysis was performed among 12 melanoma cases for classification of immunohistochemical outcome, including evaluation of staining intensity (negative, weak, moderate) and fraction of stained cells (negative, <25, 25–75 or >75%). Lumican-encoding gene (i.e. *LUM*) was assessed for mRNA expression among 44 patients with metastatic melanoma. Publicly available Bhardwaj gene expression dataset^26^ was obtained from R2 microarray analysis and visualization platform (http://r2.amc.nl). Cut-off value for separating high and low *LUM* expression groups in survival analysis was determined by the online algorithm. Among skin cutaneous melanoma dataset from The Cancer Genome Atlas (TCGA; https://tcga-data.nci.nih.gov/tcga), risk estimation was performed using SurvExpress biomarker validation tool^66^. Risk groups were generated based on prognostic index (PI) calculations (higher values for higher risk), with PI being known as the linear component of the Cox model^67^. Two groups were split from the ordered PI according to SurvExpress optimization algorithm, with chosen split point corresponding to the lowest logrank *p*-value. *LUM* gene mutation rate was also assessed in 4 datasets providing comprehensive profiling of melanoma clinical samples^27, 68, 69^. The cBio Cancer Genomics Portal^70, 71^ was used to investigate the impact of *LUM* somatic mutations on patients’ overall and disease-free survival as well as to establish gene expression correlations among TCGA data.

### Animal care

*Lum* ^−/−^ mouse line was generated by targeted mutation and fixed to the C57BL/6J genetic background (B6.129S-*Lum* ^*tm1Chak*^/J)^37^. PCR-based genotyping was performed to distinguish between homozygous *Lum* ^−/−^ mice and their wild-type (WT, i.e. *Lum* ^+/+^) littermates. A mixture of three primers (forward primer, 1893U: 5′-AAG CAG GGG ATG TTA AGC TGC-3′, reverse primers 2187: 5′-ACG TGC TAC TTC CAT TTG TCA CG-3′ and 2231L: 5′-TCA GGG TAT TTC CTG GTG GCA C-3′) were used to amplify a 338 bp and a 294 bp products from wild type and lumican deficient mice, respectively. This study was performed in compliance with “The French Animal Welfare Act” and following “The French Board for Animal Experiments”. Experiments were conducted under approval of the French “Ministère de l’Enseignement Supérieur et de la Recherche” (ethics committee n°C2EA-56) in compliance with the “Directive 2010/63/UE”.

### SDS-PAGE and Western-blot

Total protein (25 µg) were prepared from skin samples of wild type (*Lum* ^+/+^) mice (*n* = 3) and lumican knockout mice (*Lum* ^−/−^) (*n* = 3) and then loaded in Laemmli buffer. Samples were resolved by SDS-PAGE and then transferred to PVDF membrane. Lumican was identified using a rabbit antiserum generated against lumican core protein. The rabbit immunoreactive serum raised against lumican was prepared after three intra-dermal injections of lumican core protein every three weeks as previously described^15, 72^. The antiserum was used at a 1:1000 dilution and a HRP-conjugated goat anti-rabbit secondary antibody (1:10000) was considered for substrate detection with the ECL kit (Pierce, Rockford, IL).

### TAX2 peptide formulation

TAX2 peptide (CEVSQLLKGDAC) acting as a specific antagonist for TSP-1:CD47 interaction^20^ was synthesized and purified by Genecust (Dudelange, Luxembourg) and then controlled for composition and purity through electrospray ionization-mass spectrometry (ESI-MS) and HPLC. On the day of dosing, TAX2 was dissolved with injectable saline (Sigma-Aldrich, Saint-Quentin Fallavier, France) to reach a final concentration of 1 mg/ml prior to the i.p. injection (10 ml/kg). Ready-to-use vehicle was used as a control (10 ml/kg).

### Allograft model

B16F1 cell line was obtained from the American Type Culture Collection (ATCC) and maintained in culture as previously reported^20^. For allograft experiments, 2.5 × 10^5^ B16F1 cells in suspension within 100 µl of RPMI-1640 medium (Gibco, Life Technologies, Saint- Aubin, France) were s.c. inoculated into the left flank of randomized groups of either WT (*Lum* ^+/+^) or *Lum* ^−/−^ mice (*n* = 8–12 per group). Systemic (i.p.) administrations of TAX2 (10 mg/kg BW) or injectable saline (0.9% NaCl) were performed at days 3, 5 and 7. Tumor measurements and animal monitoring were performed as reported elsewhere^20^. At study termination, mice were sacrificed and tumors were surgically extracted, weighted and then fixed in 3.7% formalin.

### Histopathological analyses

Histological analyses of paraffin-embedded s.c. allografts were performed on hematoxylin, eosin and saffron (HES)-stained sections prepared using routine histological methods. Anti-TSP-1 (Abcam ab85762; 1:250 dilution), anti-CD3 (Dako A0452; 1:250), anti-VEGF (Abcam ab46154; 1:500) and anti-CD31 (Dianova DIA-310; 1:100) antibodies as well as the rabbit polyclonal antibody raised against lumican core protein^15, 72^ (1:1600) were used to perform immunostaining together with biotin-labeled secondary antibodies and streptavidin-HRP AEC (3-amino-9-ethylcarbazole) detection system (Abcam), and then followed by hematein counter-stain. Negative controls were performed by omitting the primary antibody and by using isotype-matched non-immune immunoglobulins (Dako) as negative controls. The number of functional blood vessels (i.e. vessels displaying endothelial layer integrity) and their mean diameter as well as relative CD3- and CD31-positive areas were determined in five random ×100 fields. Tumor necrotic part as well as VEGF-positive area and intratumoral blood vessels positive for TSP-1 immunostaining were quantified across the whole s.c. allograft sections. All quantitative analyses were performed using ImageJ software binarization and thresholding tools.

### µCT tumor angiography

*In vivo* µCT was performed on isoflurane-anaesthetized tumor-bearing mice at days 9 and 14 after melanoma cell inoculation. CT images were acquired on a dedicated small animal µCT scanner (Skyscan 1076, Bruker, Kontich, Belgium) while continuously rotating the camera by 180° with the following parameters: 50 kV, 0.5 mm Al filter, 200 µA source current, 35 µm isotropic resolution, 180 ms exposure time, 4 projection images per 0.7° rotation step and a retrospective synchronization. Projections were reconstructed using Skyscan software (NRecon, Skyscan) filtered backprojection algorithm. Tumor angiography was performed after retro-orbital injection (5 ml/kg BW) of the alkaline-earth metal-based nanoparticulate CT imaging agent ExiTron^TM^ nano 12000 (Viscover^TM^, nanoPET Pharma GmbH). Mice were imaged during the first 30 min post imaging agent application, a time period during which no reduction in contrast was observed^73^. Analysis of reconstructed images was performed using Amira 5.4.3 software (Visualization Sciences Group, Burlington, MA).

### Fourier transform infrared (FT-IR) microimaging

For FT-IR microimaging, 7 µm tumor sections were transferred onto CaF_2_ windows and then spectral images were collected using an infrared microscope (Spotlight 400 Imaging System, Perkin-Elmer, Courtaboeuf, France) coupled to a Frontier spectrometer. The device is equipped with a nitrogen-cooled mercury cadmium telluride 16-pixel-line detector for imaging and a computer-controlled stage to collect large spectroscopic images from tumor samples. The microscope was isolated in a venting Plexiglas chamber to enable purging dry air and to eliminate atmospheric interferences. Each pixel sampled a 6.25 × 6.25 µm^2^ area, providing images that contained 170,000 to 310,000 spectra depending on the size of sample. Therefore, acquisitions on allograft sections were split into 3 images per s.c. tumor due to software limitations and complexity of further calculations during image pre-processing and analysis. Spectral data were acquired in transmission mode. All measurements were recorded using a spectral resolution of 8 cm^−1^ and averaged to 4 scans per pixel on spectral range 800 to 4000 cm^−1^. A background spectrum was collected (120 accumulations, 8 cm^−1^ resolution) apart from the sample to ratio against the single-beam spectrum. Resulting spectra were then automatically converted into absorbance. Further analyses were performed using in-house algorithms written in Matlab (The Mathworks, Natick, MA). A modified Extended Multiplicative Signal Correction (EMSC) method was used to numerically correct the contribution of paraffin in FT-IR spectra^74^ as well as to eliminate spectra with low signal-to-noise ratio^75^.

### FT-IR images unsupervised *K*-means clustering

With the objective to detect subtle intratumoral biological differences that may not be addressed through conventional histology approaches^33, 34^, an unsupervised cluster analysis was applied to FT-IR microspectroscopy using the *K*-means method. For this purpose, we focused on spectra within the 900 to 1800 cm^−1^ IR absorption range that is considered to be the most informative region as far as the tissue biochemical features are concerned^35, 76^. Analyses were based on second derivative spectra, that can be utilized to improve the resolution of absorbance bands while maintaining a relationship to intensity of the original raw spectral absorbance. *K*-means clustering iteratively partitions spectra into different classes based on spectral signatures. First, *K* spectra (*K* is the predefined number of searched clusters) are randomly chosen to represent initial centroids which model the mean spectrum of each cluster. Second, each spectrum is affected to the cluster with the nearest centroid according to the Euclidean distance. Third, each centroid is updated as the mean of the spectra belonging to its cluster. Steps 2 and 3 are repeated until algorithm convergence is reached. The spectral distance between different *K*-means cluster centroids could be visualized *via* a dendrogram obtained by hierarchical clustering analysis using Ward’s linkage algorithm. In *K*-means, each spectrum belongs to a unique cluster and can thus be represented by a unique color distinct from those of the remaining clusters so as a color-coded image can be reconstructed and then compared to adjacent HES-stained section. This method allows rapid visual as well as quantitative analysis of clustering results reflecting tumor tissue heterogeneity.

### FT-IR collagen microimaging

Pre-processed EMSC images were then analyzed using a previously validated methodology for visualizing collagen deposition, developed with the purpose of evaluating treatment strategies involving ECM^40^. Briefly, the method consists of determining an absorbance ratio between the baselined and integrated areas of the absorbance band centered at 1338 cm^−1^ arising from collagen CH_2_ side chain vibrations^77^ and the amide I absorbance band centered near 1650 cm^−1^ mainly arising from the C=O stretching vibration of proteins^36^. This allows collagen mapping across the tumor tissue section, creating an intensity image of collagen deposition. As the 1338 cm^−1^ is a very small absorbance band, second derivative analysis of spectra was also performed to facilitate peak identification and to confirm the distribution at that wave number. For quantitative analyses, similar threshold value and color-map were applied to FT-IR images, which were then imported into ImageJ software as 8-bit gray-scale intensity images, resized and normalized before binarization so as to isolate areas corresponding to collagen deposition from the rest of the tissue. The percentage of stained pixels (collagen) within each tumor sample was measured.

### Polarized light microscopy

For studying ECM collagen organization, de-paraffinized slides were stained using Abcam picrosirius red stain kit (ab150681) according to the manufacturer’s instructions. Picrosirius staining may be viewed using polarized light resulting in birefringence of the collagen fibers and distinction between type I (thick fibers, red birefringence) and type III (thin fibers, green birefringence) collagen. Slides were imaged in dark-field using a Zeiss Axiovert 200 M microscope equipped with cross-polar optics and a CCD camera. Images from each slide were captured at ×63 magnification in 3 different locations per animal. For each microscopic field, color channels were split in ImageJ software before thresholding so as to quantify the percentage of red and green pixels as well as the corresponding type I/type III ratio. Mean red and green intensities were also calculated for each image and then averaged. To assess the basketweave structure of collagen, an innovative bioimaging approach combining Fast Fourier Transform (FFT) with Gabor filtering was applied^41^. Picrosirius red images were first converted to monochrome grey scale into ImageJ software and then a 3 × 3 median filter was applied to remove photon noise generated during image acquisition. Gabor filtering was performed using ω direction values of 45° + 225°, 90 + 270°, 135 + 315° and 0° + 180° so as to detect and highlight collagen fiber edges. Before FFT processing, windowing was performed on Gabor-filtered images in order to minimize vertical and horizontal discontinuities at the image edges, that may result in artefactual lines in the frequency domain. As FFT extracts the strength of the different frequency waveforms contributing to the pixel values of Gabor-filtered cross-polar collagen images, elliptical measurements of the scatter pattern for each orientation may be used to determine a collagen orientation index.

### Second harmonic generation imaging

Collagen SHG imaging was performed using a Zeiss multiphoton laser-scanning LSM710 NLO microscope equipped with a ×20 objective (0.8 NA). A titanium:sapphire laser (Coherent Inc., Santa Clara, CA) tuned to 860 nm provided the illumination light, while emitted photons were detected through a 430 ± 20 nm filter. Single-frame images of 3 different locations for each tumor were acquired. Collagen density was quantified as the sum of the thresholded image, which represents the portion of collagen-positive pixels. The frequency of angles were plotted in polar coordinates using SurfCharJ plugin^78^ so as to indicate the preferred orientation of collagen fibers within tumor tissue.

### Statistical analyses

Significance for Kaplan-Meier overall and disease-free survival analyses were assessed by logrank test. Spearman’s *r* coefficient was calculated to estimate correlation between different genes expression as well as between allograft tumor volume and collagen orientation index. Comparison between two groups was performed using Student’s *t* test, and histograms display data as mean ± SEM. For *in vivo* experiments, groups were compared with the non-parametric Mann-Whitney *U* test for unpaired samples using Prism 5.0 (GraphPad Software, La Jolla, CA, USA). Two-sided *p* values < 0.05 (*), <0.01 (**) or <0.001 (***) are indicated when statistical significance is reached.

## Electronic supplementary material

Supplementary Information
