# Supplementary material for: Lumican delays melanoma growth in mice and drives tumor molecular assembly as well as response to matrix-targeted TAX2 therapeutic peptide
Source: Sci Rep. 2017 Aug 9;7:7700. doi: 10.1038/s41598-017-07043-9 (PMC5550434; doi:10.1038/s41598-017-07043-9)
Supplement: Supplementary file 1 — Supplementary Information [file 41598_2017_7043_MOESM1_ESM.pdf]

Lumican delays melanoma growth in mice and drives tumor molecular assembly as well as response to matrix-targeted TAX2 therapeutic peptide.

Albin Jeanne[1,2,3], Valérie Untereiner[2,4], Corinne Perreau[2,5], Isabelle Proult[2,5], Cyril Gobinet[2,6], Camille Boulagnon-Rombi[2,7], Christine Terryn[4], Laurent Martiny[1,2], Stéphane Brézillon[2,5] and Stéphane Dedieu[1,2]*.

SUPPLEMENTARY INFORMATION

Supporting information captions

Fig S1. *COL1A2* and *MMP14* are co-expressed with *LUM* in human melanoma and both correlate with outcome.

(**a**) *COL1A2* and *MMP14* encoding genes were retrieved within *LUM* interaction network queried from the Human Protein Reference Database HPRD[79]. Heatmap depicts relative expression of *LUM*, *COL1A2* and *MMP14* genes in cutaneous melanoma *vs*. normal skin, revealed using Talantov dataset[80] from Oncomine DB[81]. (**b-c**) High *COL1A2* mRNA (**b**) and low *MMP14* mRNA (**c**) expression is associated with improved survival in patients with melanoma. Kaplan-Meier analyses for overall survival rates of 44 melanoma patients were performed among Bhardwaj dataset[26] (GEO accession number GSE19234) using the R2 web tool as described in Materials and Methods.

Fig S2. Analysis of normal skin features in *Lum*$^{+/+}$ (WT) *vs. Lum*$^{-/-}$ mice.

(**a**) Representative micrographs (×20) of HES-stained sections through contralateral skin surfaces of *Lum*$^{+/+}$ (WT) *vs*. *Lum*$^{-/-}$ mice. Note skin thickening in *Lum*$^{-/-}$ mice which is associated with a deeper dermis layer while epidermis becomes thinner (*black double arrow*). Lumican-null phenotype also correlates with larger and disorganized collagen fibers (*blue arrows*). (**b**) Representative polarized light micrographs, using crossed polars, of picrosirius red-stained normal skin sections (original magnification ×63). (**c**) Representative collagen SHG images (original magnification ×20). (**d-f**) Quantification of the relative distribution of (**d**) red (type I collagen fibers) and (**e**) green (type III collagen fibers) pixels within normal skin picrosirius-stained images, and corresponding (**f**) averaged type I/type III ratio calculations (mean ± SEM, *t* test, ns not significant). (**g**) Collagen orientation index determination from images of picrosirius-stained polarized images of normal skin processed through Gabor filtering and FFT (mean ± SEM, *t* test, *** $p < 0.001$). (**h**) Collagen density of skin SHG images for *Lum*$^{+/+}$ *vs*. *Lum*$^{-/-}$ animals (mean ± SEM, *t* test, ns not significant). (**i**) Representative polar plots of SHG intensity *vs*. angle of laser polarization.

Fig.S1

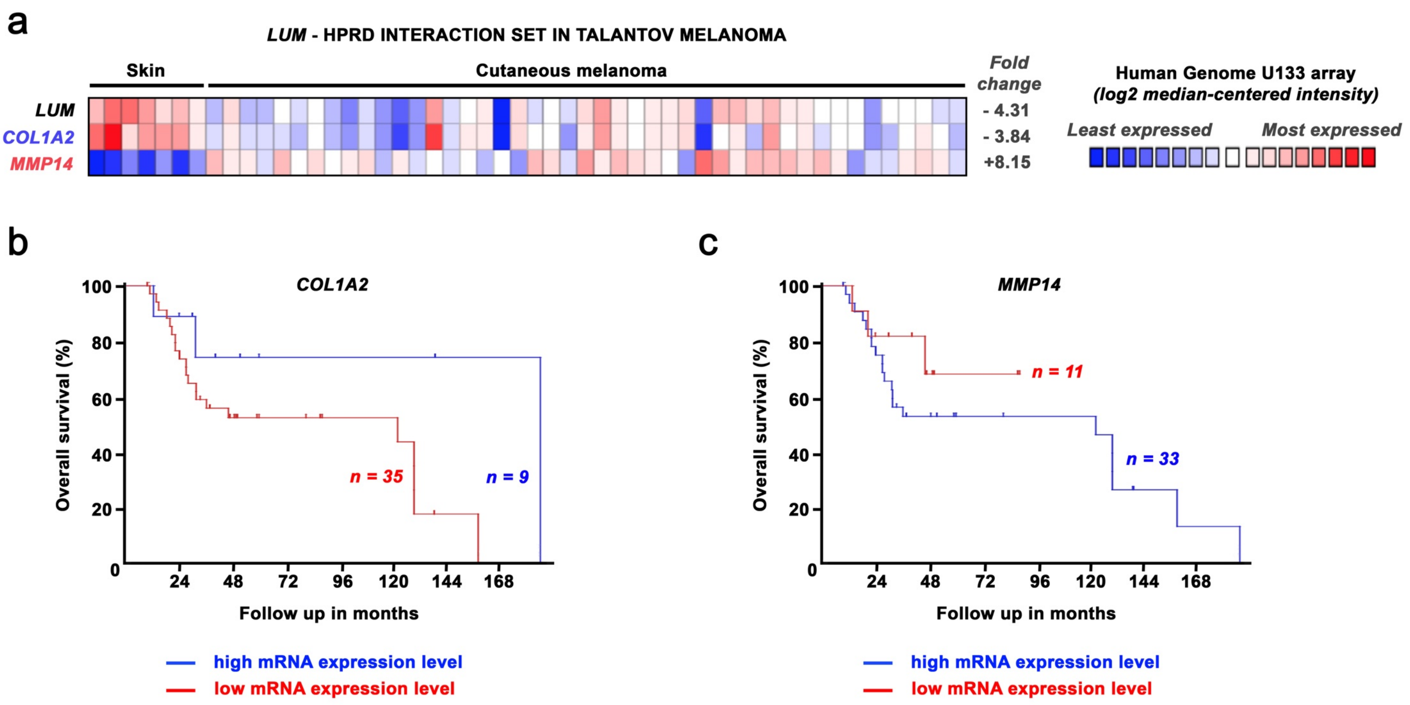
a
LUM - HPRD INTERACTION SET IN TALANTOV MELANOMA
Skin
Cutaneous melanoma
Fold change
LUM
COL1A2
MMP14
- 4.31
- 3.84
+8.15
Human Genome U133 array
(log2 median-centered intensity)
Least expressed
Most expressed
b
COL1A2
Overall survival (%)
100
80
60
40
20
0
24
48
72
96
120
144
168
Follow up in months
n = 35
n = 9
high mRNA expression level
low mRNA expression level
c
MMP14
Overall survival (%)
n = 11
n = 33
Follow up in months
high mRNA expression level
low mRNA expression level

Fig.S2

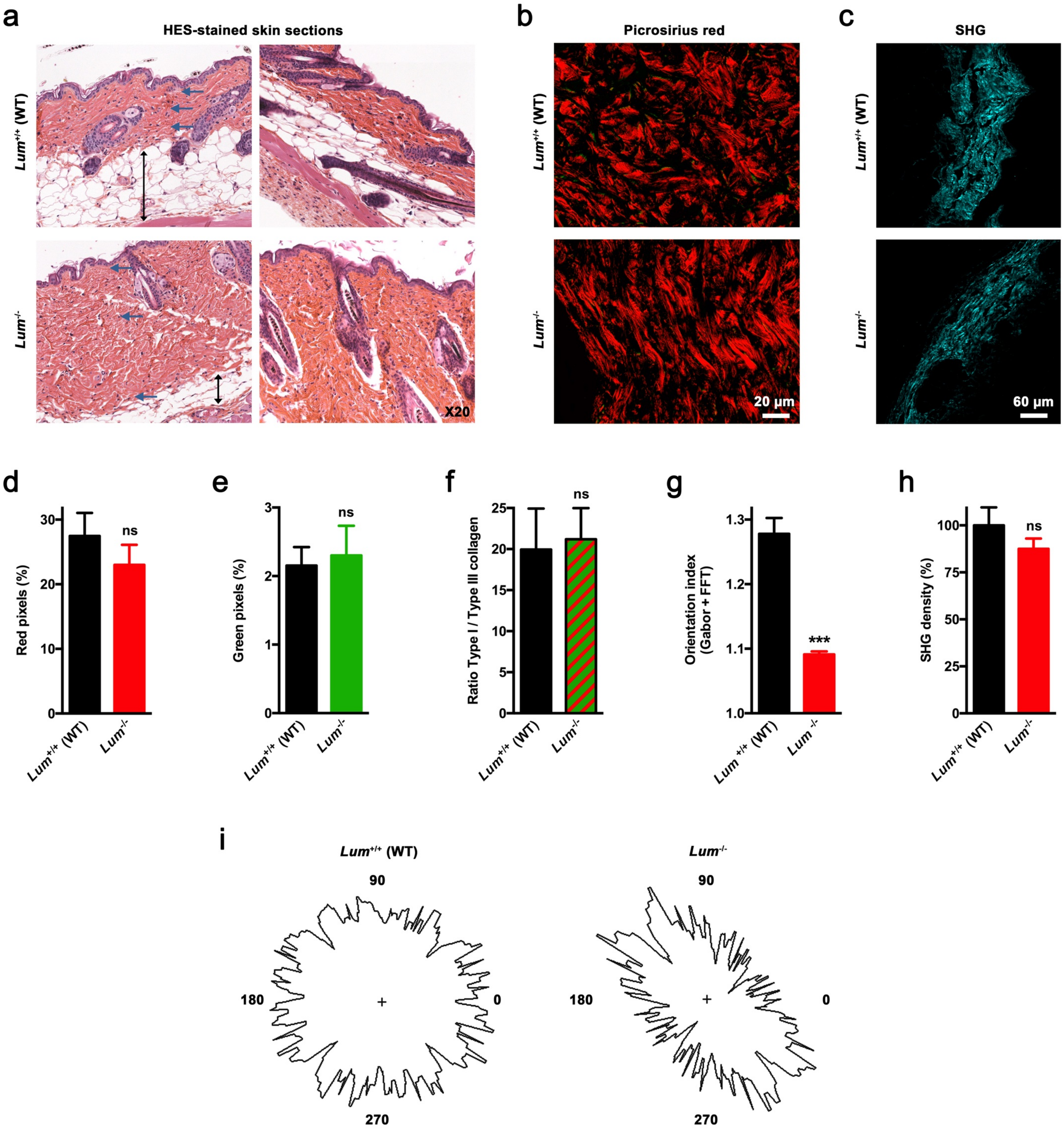

a
HES-stained skin sections
Lum+/+ (WT)
Lum-/-
X20
b
Picrosirius red
Lum+/+ (WT)
Lum-/-
20 µm
c
SHG
Lum+/+ (WT)
Lum-/-
60 µm
d
Red pixels (%)
ns
Lum+/+ (WT)
Lum-/-
e
Green pixels (%)
ns
Lum+/+ (WT)
Lum-/-
f
Ratio Type I / Type III collagen
ns
Lum+/+ (WT)
Lum-/-
g
Orientation index
(Gabor + FFT)
***
Lum +/+ (WT)
Lum -/-
h
SHG density (%)
ns
Lum+/+ (WT)
Lum-/-
i
Lum+/+ (WT)
Lum-/-
90
180
0
270

References

79. Keshava Prasad, T. S. *et al.* Human Protein Reference Database-2009 update. *Nucleic Acids Res.* **37**, D767-D772 (2009).

80. Talantov, D. *et al.* Novel genes associated with malignant melanoma but not benign melanocytic lesions. *Clin. Cancer Res.* **11**, 7234-7242 (2005).

81. Rhodes, D. R. *et al.* Oncomine 3.0: genes, pathways, and networks in a collection of 18,000 cancer gene expression profiles. *Neoplasia* **9**, 166-180 (2007).
